# Pattern of NSAID Poisoning in a Referral Poisoning Center of Iran: Solutions to Reduce the Suicide

**DOI:** 10.22037/ijpr.2019.111720.13316

**Published:** 2019

**Authors:** Nazanin Bagherian Rad, Mitra Rahimi

**Affiliations:** a *Department of Toxicology and Pharmacology, Islamic Azad University of Medical Sciences, Tehran, Iran. *; b *Department of Clinical Toxicology, Loghman-Hakim Hospital, School of Medicine, Shahid Beheshti University of Medical Sciences, Tehran, Iran.*

**Keywords:** Anti-inflammatory agents, Non-steroidal, Poisoning, Adverse effects, Overdose

## Abstract

NSAIDs are nonsteroidal anti-inflammatory drugs, thus, they will provide analgesic, antipyretic, anti-inflammatory, antiplatelet effects. Severe poisoning and death because of acute intoxication can occur by ingestions of more than 400 mg/kg. This cross-sectional retrospective study was carried out in on all patients referred to Loghman Hakim Hospital from 2011 to 2016. Grouping of our patients was based on the amount of NSAID ingestion, Type of NSAID, patient’s conscious level according to Reed Scaling criteria, suicide attempt, and gender. Data were analyzed using the SPSS software. A *P*-value of 0.05 or less was considered to be statistically significant. The period prevalence of NSAID poisoning was 0.14% and the incidence was 3.816/100,000/year. The uppermost number of poisoning were seen in 2012 (20.96%). Mean age was 23.75 ± 9.76 years and most of the intoxications were seen in females (66.37%). Of the patients, 140 (61.13%) had ingested less than 200 mg/kg, and 9.17% committed suicide having a mortality rate of 0.43%. The most common NSAIDs that had been used were Ibuprofen (73.79%). Of patients, 83.4% underwent through common complications of NSAID poisoning. We find significant relationship between the type of NSAID and higher sodium, BUN, ALT, ALP, CPK levels in men, and higher LDH level in women. Besides, we found substantial correlation between using short-acting NSAIDs and female gender, suicide action, arrival to the hospital less than 12 h, amounts under 200 mg/kg, hospitalization longer than 12 h, and presentation of loss of consciousness.

## Introduction

NSAIDs are nonsteroidal anti-inflammatory drugs. They, non-selectively, impede cyclo-oxygenases and reduce the formation of prostaglandins and thromboxanes (Eicosanoids in cell membranes). Thus, they will provide analgesic, antipyretic, anti-inflammatory, anti-platelet effects. This group of drugs has one prototype named Acetylsalicylic acid (ASA) that itself consists of over 20 types of drugs and irreversibly hinder cyclooxygenases (1). After ingestion, NSAIDs mostly are gone through hepatic metabolism by CYP2C8, 2C9, 2C19 and/or glucuronidation. They tend to bind to proteins, which define their distribution to the central nervous system (CNS). The metabolites excrete via glomerular filtration and tubular discharge. NSAID’s half-life is either short acting (<8 h) or long acting (>8 h). Consequently, active metabolites of those with longer half-life would have further enterohepatic circulation (2–3). Severe poisoning and death as a result of acute intoxication with NSAIDs can occur by ingestions of more than 400 mg/kg. There are six aspects of acute NSAID toxicity (4). First and following the intake of great amounts of NSAIDs such as ibuprofen, naproxen, and phenylbutazone, enhanced anion gap metabolic acidosis take place. This can cause cardiac dysrhythmias and electrolyte deviations (5–8). Acute renal failure (reversible) is the second clinical aspect of acute NSAID toxicity that is due to vasodilator prostaglandins’ blockage (9). Third and in cases of ibuprofen overdose, hypotension and cardiovascular collapse cause cardiovascular toxicity (8). Moreover, 30 percent of Ibuprofen intoxications would develop CNS toxicity (drowsiness to coma) (10). Yet seizure was observed only with intake of propionic acids, pyrazolones, acetic acids, and anthranilic acids (11–13). Besides after ingestion of phenylbutazone and indomethacin aplastic anemia and agranulocytosis have been reported. Last but not least, anaphylaxis has been shown in certain cases as well (14). The 2016 report of the American Association of Poison Control Centers, demonstrated that analgesics are still the most common category of drug in acute overdose (11.2%; 18.1% intentional overdose; 80.42% fatality following ingestion) having slightly increase over the past few years (15). We found the same results from the National Poisons Information Service of United Kingdom with paracetamol in the first place and ibuprofen in the second (16). Although there are several case reports from other countries, we find no report on NSAIDs toxicity’s incidence, prevalence, distribution, and mortality in Iran. Therefore, the main aim of this study is an epidemiological investigation on NSAIDs intoxication, along with finding solutions to reduce the suicide actions associated with NSAID abuse.

## Experimental

After confirmation by the Ethics Committee of Islamic Azad University of Medical Sciences, this cross-sectional retrospective study was carried out in on all patients referred to Loghman Hakim Hospital from January 2011 to January 2016. This center is a referral poison center, which serves a population of more than 12.5 million and has an annual emergency department census of 24000 to 30000 only for poisoned patients. Based on the Loghman Hakim Hospital of Shahid Beheshti University of Medical Sciences’ policies, written consent is given for using patient’s information for research prior to their admission to the hospital. In addition, the study protocol obeys the ethical guidelines of the Declaration of Helsinki. Entry criteria include patient’s complete record, a definitive diagnosis of pure NSAID toxicity. A self-made questionnaire containing information regarding the demographics, type of NSAID and purpose of use, on-presentation vital signs and laboratory data, duration of hospitalization, treatments given, and the outcome was filled for every single patient. At large, a total of 14,754 patients with NSAIDs poisoning and consumption of simultaneous drugs were extracted from hospital’s database. Only 229 cases had pure NSAID toxicity during six-year period. Grouping of our patients was based on the amount of NSAID ingestion (below 200 mg/kg; 200-400 mg/kg; more than 400 mg/kg), Type of NSAID (short *vs.* long acting NSAIDs), patient’s conscious level according to Reed Scaling criteria (loss of consciousness or LOC and no loss of consciousness or No LOC), suicide attempt, and gender. It should be noted that short-acting NSAIDs are Ibuprofen, Diclofenac, Indomethacin, Aspirin, Mefenamic acid that have less than eight hours half-life. In contrast, there are long acting NSAIDs, which are as follows: Naproxen, Celecoxib, Meloxicam, and Piroxicam. Data were analyzed using the social package for statistical analysis SPSS software version 25. The data was presented by mean ± SD for continuous variables and frequency for categorical variables. Distribution of the data was tested by the Kolmogorov–Smirnov test. The Chi-square (plus odds ratio) and Fisher’s exact tests along with Lambda, Phi, Cramer’s V., Eta, Pearson, Spearman correlation coefficients were used to analyze qualitative variables. Statistical comparison was made using Mann–Whitney U-test or Kruskal-Wallis test for nonparametric variables and independent t-test (Two-tailed) or One-way Anova for parametric ones. Multiple Regression (linear) was used to foresee the value of a variable based on the value of other variables. The outcome (dependent variable) was type of the NSAID that caused poisoning. The Predictor (independent variable) were demographics, vital signs, arterial blood gases analysis, laboratory results, and complications. For all tests, a *P*-value of 0.05 or less was considered to be statistically significant.

## Results

Here, we present the number of clients referring to the emergency department of Loghman Hakim hospital with pure NSAID poisoning in six years. The period prevalence of NSAID poisoning was 0.14% in six years, and the incidence was 3.816/100,000/year. It should be noted that total population of hospitalized poisoned patients within these six years in our hospital was 164,333.

All of the 229 participants had oral ingestion of NSAIDs. The uppermost number of NSAID poisoning were seen in 2012 (n = 48, 20.96%), and the lowermost number of whom were related to the year 2015 (n = 29, 12.66%). Other year’s distribution of NSAID intoxicated patients was as follows: 2011 (n = 38, 16.59%), 2013 (n = 44, 19.21%), 2014 (n = 30, 13.10%), 2016 (n = 40, 17.46%).

Based on the Demographics results, the majority of patients were female 66.37% (152 cases). Mean age of patients was 23.75 ± 9.76 years with a range of 13 to 90 years. With respect to gender, the average age was 25.09 ± 9 and 23.08 ± 9 for males and females, correspondingly. Of the patients, 140 (61.13%) had ingested less than 200 mg of NSAIDs per kg, 59 cases (25.76%) had consumed 200 to 400 mg/kg, and 30 cases (13.10%) had ingested more than 400 mg/kg of NSAIDs. Twenty-one individual has committed suicide action and the mortality rate of our study was 0.43%. 

Of the included patients, 224 cases (97.81%) had intentional consumption and the rest (2.18%) of our patients had used NSAIDs inadvertently. The NSAIDs that had been used were Ibuprofen (n = 169), Aspirin (n = 36), Diclofenac (n = 3), Naproxen (n = 3), Mefenamic Acid (n = 3), Indomethacin (n = 2), Meloxicam (n = 1). Moreover, there were cases who used combination of NSAIDs [Aspirin + Ibuprofen (n = 3), Ibuprofen + Indomethacin (n = 2), Ibuprofen + Diclofenac (n = 2), Ibuprofen + Naproxen (n = 1), Ibuprofen + Mefenamic acid (n = 3), Ibuprofen + Diclofenac + Naproxen + Celecoxib (n = 1)]. 

Table 1 shows general characteristics of NSAID poisoned patients from their arrival to the emergency department to their release from the hospital. All of which revealed to have a significant correlation with using short acting NSAIDs. In addition, through analysis of the time interval (hours) between NSAID ingestion and arrival of poisoned patients to the hospital revealed that most of the subjects arrived under 4 h (n = 139, 60%), and the minority of patients arrived between 17-20 h interval (n = 3, 1%).

**Figure 1:**
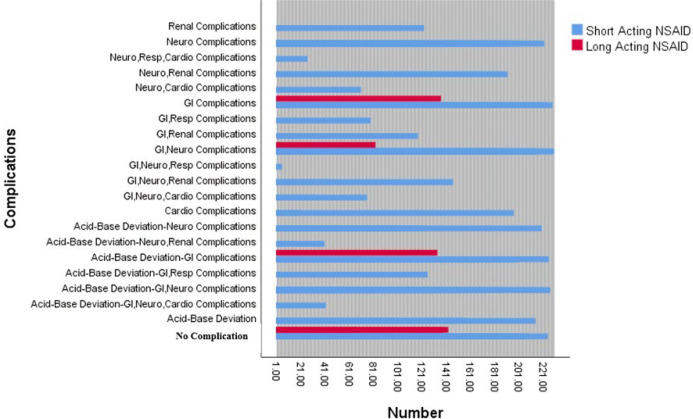
Complications of NSAID poisoned individuals

On the basis of clinical manifestations, 191 patients underwent through common complications of NSAID poisoning (Figure 1). The majority of our patients presented gastrointestinal complications (n = 125, 65.44%), followed by neurologic complications (n = 12, 62.82%) and acid-base deviations (n = 55, 28.79%). On the other hand, respiratory complications were only observed in four cases (2.09%). 

Based on every patient’s condition, we used different therapeutic approaches. Commonly, the most prevalent treatment option was mixture of administration of activated charcoal and sorbitol, fluid therapy, psychiatric consult, and supportive treatment (76.4%). All of our patients were undergone supportive treatment and fluid therapy, and 227 (99.12 %) of whom were administrated with charcoal and sorbitol. Of patients, 226 cases (98.68%) had psychiatric consult. The smallest amount of individual treatment approaches were belong to bicarbonate sodium administration (7 cases, 3.05%), and gastrointestinal washing (6 cases, 2.62%). In addition, eight (3.49%) patients required to be intubated, and consequently they were transferred to the intensive care unit.

Unfortunately, one of our patients did not survive. Patient was female with 16 years of age. She took Ibuprofen orally, having intentional purpose of use but did not commit suicide. The amount of drug was under 200 mg/kg. She was arrived to the emergency room of hospital having 1-4 hinterval between ingestion and entrance. Her on arrival vital stats were as follows: PR = 120 per min, RR = 18 per min, BP = 85/50 mmHg, T = 37 °C. Her consciousness level was two on the basis of reed scale criteria. ABG analysis revealed respiratory acidosis (pH 7.24, PCo2 = 66.70, HCo3 = 16.60) (Table 2). Treatment options were administration of activated charcoal and sorbitol, gastrointestinal washing, fluid therapy, intubation, bicarbonate sodium, and supportive care. Ultimately, she underwent neurological (agitation and seizure), renal complications (acute renal failure followed by vasodilator prostaglandin’s blockage), and acid-base deviations. Despite our very best effort, she was passed away under 12 h of hospitalization. The cause of death was cardiac arrest and CPR was not successful.

## Discussion

In this study, we presented epidemiology of NSAID poisoning along with the demographics, laboratory analysis, clinical complications, and treatment options. Within six years of this study, we found 229 cases of NSAID intoxications. The majority of patients were found in 2012 year. In this study, we found a slight increase in NSAIDs abuse between 2011 and 2016. Based on CDC report, due to the aging population, we would encounter increase in using NSAIDs. Most of our intoxicated patients were female, abused ibuprofen, ingested under 200 mg/kg of NSAIDs, took under four hours between oral ingestion and emergency room admission, and were not suicidal. Of cases, 188 ones underwent major complications and one patient did not survive.

Here, the mortality rate associated with NSAID poisoning was estimated as 0.43%. It was due to neurological (agitation and seizure), renal complication, and persistent acid-base deviations followed by cardiac arrest. This result is very low, in contrast to 2016 report of the American Association of Poison Control Centers (80.42%) (15). Another study revealed a 30-day mortality of 25% resultant from peptic ulcer perforation among 2061 patients of three Danish counties (17). Besides, a systematic review study between years 1997-2008 demonstrated 61,067 cases of NSAID poisoning and revealed that the mortality rates were decreasing from 11.6% to 7.4%. This study only considered upper gastrointestinal bleed and perforation in hospitalized cases (18). One other study showed a mortality rate of 21.99% due to cardiovascular disease in United Kingdome Norfolk Arthritis Register (19). Out of these investigations we can conclude that our mortality rate in a referral poison center of Iran is lower than any other studies. This is because we considered all of the cases of NSAID poisoning, no matter what was the complication that resulted death, as long as they had used NSAIDs only. Furthermore, we included every person with any background disease that resulted their usage of NSAID.

Every day and all around the world, more than 30 million of people are using NSAIDs. Based on recent analysis on global sales, ibuprofen, diclofenac, and naproxen have the majority of sales, correspondingly either by prescription or over-the-counter (20). This can justify why Ibuprofen is among the most abused NSAIDs both in this study and many others (21). In contrast, COX-2 selective agents such as meloxicam and celecoxib are experiencing a huge decrease (55.1 to 29.2% in US; 8.4% in Germany) over the past few years, while non selective NSAIDS are having an increase globally (50.2 to 73.9% in US; 19.0 million in defined daily doses in Germany), studies in the US and Germany revealed (22, 23). In addition, although many estimations pointed 10-40% of NSAIDs usage happened in patients older than 65 years of age (21), only two cases (0.87%) of our study had this age range. This reveals that our young population had more inclination toward using NSAIDs than elderly patients. The reasons could be this young population is not aware of potential side effects of NSAIDs mainly because they are bought over-the-counter and they are presumed to be safe.

There are many reports on complications associated with NSAID abuse. The incidence of gastrointestinal complications (both upper and lower GI) is 12.9-50.4% based on systemic reviews (20). However, the incidence of GI complications in our study was higher (65.44% of adverse effects, 54.58% of total included population). It was seen, particularly, in cases who used ibuprofen, naproxen, aspirin, diclofenac, mefenamic acid, and indomethacin. Studies showed that COX-2 selective NSAIDs have lower risk for GI complications than others. It should be noted that there are several risk factors pertaining to NSAIDs’ GI complications, such as higher dosage use, elderly, *Helicobacter Pylori* infection, and history of peptic ulcers. Likewise, simultaneous use of low-dose aspirin can magnify GI adverse effects (20). 

Neurologic complications consisted 62.82% of the complications of our study. Ibuprofen, aspirin, indomethacin, meloxicam, and mefenamic acid were among the culprit NSAIDs in such patients. Based on a prospective study, 30% of cases with ibuprofen overdose developed CNS toxicity (10). Seizures have been reported with abuse of ibuprofen, diclofenac, indomethacin, and mefenamic acids both in large and small doses. Seizures could be due to decline in production of prostaglandin and thromboxane (11). 

Acid-base alteration had the third grade of adverse effects in our study (28.79%). It was seen in patients who ingested ibuprofen, indomethacin, aspirin, naproxen, and diclofenac. Studies indicated that metabolic acidosis can occur in such patients due to the enhanced onion gap, and it is especially associated with ibuprofen and naproxen (5–8). In this study, 18 (32.72%) of cases with acid-base abnormalities experienced metabolic acidosis, nonetheless, most of the cases underwent through respiratory acidosis (n = 27, 49.09%).

Laboratory analysis for detection of NSAID poisoning consist of BUN and creatinine, serum electrolytes, complete blood count, and arterial blood gasses (24). In this research, we find significant relationship the NSAID type and the following laboratory values; A) Elevated values in males: 1. Na (141.58 ± 3.75, 0.06 mmol/L increase in men compared to women), 2. BUN (32.80 ± 11.10, 8.78 mg/dL increase in men compared to women), 3. ALT (24 ± 23, 6 U/L increase in men in men compared to women), 4. ALP (219 ± 136, 29 U/L increase in men compared to women), 5. CPK (243 ± 507, 140 µg/L increase in men compared to women). B) Elevated values in females: LDH (498 ± 178, 14 U/L increase in women compared to men). Although, there are scarce amount of studies that were reported laboratory values, in NSAID poisoned patients, we found one case report that represent same results with respect to laboratory panels only, as ours (25). 

Since there are no antidotes for NSAID poisoning, management of such cases is to secure airways, decontamination of GI tract, correction of acid-base deviations, using benzodiazepines for seizures (26).

In conclusion, we find significant association between the type of NSAID and higher sodium, BUN, ALT, ALP, CPK levels in men, and higher LDH level in women. In addition, we found substantial correlation between using short-acting NSAIDs and female gender, suicide action, arrival to the hospital less than 12 h, amounts under 200 mg/kg, hospitalization longer than 12 h, and presentation of loss of consciousness.

According to the results of this study, a part of NSAIDs poisoned individuals used NSAIDs intentionally and suicidally. The proper use of media advertising, holding various seminars as well as conducting more research on psychological problems can reduce suicide rates in our country. We recommend more investigations on psychological injuries which have a significant role in identifying root causes of suicide action. Early diagnosis of these problems could prevent mental crises lead to suicide. Moreover, adequate training and supervision of pharmacies is important for controlling NSAIDs poisoning. Because these are over-the-counter drugs (OTC), they are being sold without prescription. The consequences are easier access for people, negligence the side effects, and misuse of the drug for non-therapeutic purposes. Finally, studies with a larger sample size and across the country can provide a wealth of information on the situation and deliver patterns of consumption in the country. Besides, timely diagnosis and treatment of these patients can reduce mortality and complications.

**Table 1:** General characteristics of NSAID poisoned patients

Variable	**Long-acting NSAIDs** **N = 6, Percent of Column**	**Short-acting NSAIDs** **N = 223, Percent of Column**	***P-*** **value** ^*^	**Odds Ratio**	**95% Confidence Interval**	**Multiple Regression** **R, R square, ** ***P***
Gender Male Female	3 (50%) 3 (50%)	74 (33.18%) 149 (66.81%)	<0.001	.000	0.098-2.000	R = 0.231 R^2^ = 0.053 *P* = 0.015
Year 2011 2012 2011 2014 2015 2016	0 2 (33.33%) 0 4 (66.66%) 0 0	38 (17.04%) 46 (20.62%) 44 (19.73%) 26 (11.65%) 29 (13%) 40 (17.93%)	.003	NS^**^		
Suicide Attempt	1 (16.66%)	20 (8.96%)	<0.001	<0.001	0.055-4.000	
Interval between consumption and admission <12 h >12 h	5 (83.33%) 1 (16.66%)	204 (91.47%) 19 (8.52%)	0.038	NS		R = 0.063 R^2^ = 0.004 *P *= 0.828
Amount of NSAID (mg/kg) <200 mg/kg 200-400 mg/kg >400 mg/kg	5 (83.33%) 0 1 (16.66%)	135 (60.53%) 59 (26.45%) 29 (13%)	<0.001	NS		
Duration of Hospitalization <12 h 12-24 h 24-48 h >48 h	1 (16.67%) 4 (66.66%) 1 (16.67%) 0	5 (2.24%) 209 (93.72%) 6 (2.69%) 3 (1.34%)	0.031	NS		
Presentation of Clinical Complications	3 (50%)	188 (84.30%)	0.026	NS		R = 0.152 R^2 ^= 0.023 *P* < 0.001
Loss of Consciousness	0	19 (8.52%)	<0.001	NS		
ICU Stay	0	8 (3.58%)	<0.001	NS		

^*^A *P*-value of 0.05 or less was considered to be statistically significant. ^**^NS = not significant. *P*-values are obtained using Lambda, Phi, Cramer’s V., Chi-Square, Eta, Fisher’s Exact, Pearson, Spearman correlation coefficients based on each variable.

**Table 2:** The on arrival Vital Signs, Arterial Blood Gases, Laboratory Panel distribution, and age in the included patients of this study

**Parameter**	**Gender**				***P*** **-value** ^**^	***Suicide***				**P-** ***value***	***Multiple Regression*** **R, R2, ** ***P***
	**Male**		**Female**			**Yes**		**No**			
	**Mean**	**SD**	**Mean**	**SD**		**Mean**	**SD**	**Mean**	**SD**		
Age (year)	25.09	9.75	23.08	9.72	NS^***^	23.52	7.04	23.78	10	NS	*NS*
***Vital Signs***											
PR (beat/min)	92	18	90	15	NS	93	16	90	16	NS	R = 0.144 R^2^ = 0.021 P *= 0.883*
RR (beat/min)	16	3	16	2	NS	17	5	16	2	NS	
SBP (mmHg)	117	16	112	14	0.006	117	24	114.03	13	NS	
DBP (mmHg)	75	9	72	8	0.008	72	11	73	9.01	NS	
T (°C)	36	0.00	36	0.00	NS	36	0.00	36	0.00	NS	
***Arterial Blood Gases***											
PH	7	0.06	7	0.05	NS	7	0.05	7	0.06	NS	R = 0.043 R^2^ = 0.002 P *= 0.950*
PCO2	44	7	40	7	0.001	37	8.03	42	7	0.020	
HCO3^*^	25	4	23	4	0.003	21	3	24	4	0.001	
**Laboratory Panels**											
Bs (mg/dL)	114	51	111.06	18	NS	105	12	112	34	NS	R = 0.144 R^2^ = 0.021 P *= 0.883*
FBS (mg/dL)	105	41	96	32	NS	122	65	96	30	0.007	
Na^* ^(mmol/L)	141.58	3.75	141.52	3.66	<0.001	140.95	3.86	141.61	3.67	<0.001	R = 0.073 R^2^ = 0.005 P *= 0.582*
K (mmol/L)	4.12	0.40	4.17	0.39	NS	4.21	.38	4.15	.39	NS	
BUN (mg/dL)	32.8	11.1	24.02	6.6	<0.001	27	10.4	27.02	9.29	NS	R = 0.158 R^2 ^= 0.025 P *= 0.066*
Cr (mg/dL)	1.13	11.1	0.93	0.18	<0.001	1.11	.27	0.99	0.19	0.041	
AST (U/L)	29	27	24	11	NS	24	8	26	19	NS	R = 0.062 R^2^ = 0.004 P *= 0.846*
ALT (U/L)	24	23	18	10	0.003	19	14	20	16	NS	
ALP (U/L)	219	136	190	78	<0.001	196	73	200	105	NS	
CPK (µg/L)	243	507	103	83	NS	157	154	151.03	321	NS	R = 0.256 R^2^ = 0.065 P *= 0.004*
LDH (U/L)	484	202	498	178	NS	504	154	492	189	NS	
Bili T (mg/dL)	0.76	0.49	0.61	0.29	NS	0.46	0.25	0.68	0.38	0.006	R = 0.041 R^2^ = 0.002 *P *= 0.845
Bili D (mg/dL)	0.23	0.21	0.19	0.11	NS	0.14	0.07	0.21	0.16	0.016	

PR: Pulse rate; RR: respiratory rate; SBP: systolic blood pressure; DBP: diastolic blood pressure; T: temperature; Bs: blood sugar; FBS: fasting blood sugar; Na: sodium; K: potassium; BUN: Blood Urea Nitrogen; Cr: creatinine; AST: aspartate aminotransferase; ALT: alanine aminotransferase; ALP: alkaline phosphatase; CPK: Creatine phosphokinase; LDH: Lactate dehydrogenase; SD: standard deviation; Bil T: Bilirubin Total; Bil D: Bilirubin Direct. ^*^Na and HCo3 had normal distribution, thus independent sample *t*-test was used for these parameters only. ^**^A *P*-value of 0.05 or less was considered to be statistically significant. ^***^NS: not significant.
